# Multiscale modeling of mucosal immune responses

**DOI:** 10.1186/1471-2105-16-S12-S2

**Published:** 2015-08-25

**Authors:** Yongguo Mei, Vida Abedi, Adria Carbo, Xiaoying Zhang, Pinyi Lu, Casandra Philipson, Raquel Hontecillas, Stefan Hoops, Nathan Liles, Josep Bassaganya-Riera

**Affiliations:** 1 Nutritional Immunology and Molecular Medicine Laboratory (www.nimml.org), Virginia Bioinformatics Institute, Virginia Tech, 1015 Life Science Circle, 24061 Blacksburg, VA, USA; 2 Center for Modeling Immunity to Enteric Pathogens, Virginia Bioinformatics Institute, Virginia Tech, 1015 Life Science Circle, 24061 Blacksburg, VA, USA; 3 BioTherapeutics Inc., 1800 Kraft Drive, Suite 200, Blacksburg, VA 24060, USA

**Keywords:** Computational biology, Systems biology, multiscale modeling, Agent-based modeling

## Abstract

Computational modeling techniques are playing increasingly important roles in advancing a systems-level mechanistic understanding of biological processes. Computer simulations guide and underpin experimental and clinical efforts. This study presents ENteric Immune Simulator (ENISI), a multiscale modeling tool for modeling the mucosal immune responses. ENISI's modeling environment can simulate *in silico *experiments from molecular signaling pathways to tissue level events such as tissue lesion formation. ENISI's architecture integrates multiple modeling technologies including ABM (agent-based modeling), ODE (ordinary differential equations), SDE (stochastic modeling equations), and PDE (partial differential equations). This paper focuses on the implementation and developmental challenges of ENISI. A multiscale model of mucosal immune responses during colonic inflammation, including CD4+ T cell differentiation and tissue level cell-cell interactions was developed to illustrate the capabilities, power and scope of ENISI MSM.

**Background:**

Computational techniques are becoming increasingly powerful and modeling tools for biological systems are of greater needs. Biological systems are inherently multiscale, from molecules to tissues and from nano-seconds to a lifespan of several years or decades. ENISI MSM integrates multiple modeling technologies to understand immunological processes from signaling pathways within cells to lesion formation at the tissue level. This paper examines and summarizes the technical details of ENISI, from its initial version to its latest cutting-edge implementation.

**Implementation:**

Object-oriented programming approach is adopted to develop a suite of tools based on ENISI. Multiple modeling technologies are integrated to visualize tissues, cells as well as proteins; furthermore, performance matching between the scales is addressed.

**Conclusion:**

We used ENISI MSM for developing predictive multiscale models of the mucosal immune system during gut inflammation. Our modeling predictions dissect the mechanisms by which effector CD4+ T cell responses contribute to tissue damage in the gut mucosa following immune dysregulation.

## Introduction

This paper presents ENISI, a multiscale agent-based modeling platform for computational immunology. ENISI is the first agent-based modeling platform targeting enteric mucosal immune systems and capable of integrating multiple modeling techniques such as ODE, ABM, and PDE.

### Computational modeling in immunology

Computing technologies are playing increasingly important roles in immunological research. Computational models can accelerate the knowledge discovery process through effective utilization of techniques from mathematics, computer science as well as engineering. *In silico *experimentation and model analysis such as visual and data analytics enable novel computational hypothesis generation that guide wet-lab experimentation, thereby accelerating the generation of new knowledge. Traditionally, researchers develop small and domain-specific models adopting reductionist approaches. These meticulously constructed models could have great amount of details; however, they are often single scale (ex: gene regulation, signaling, etc.) and use only one type of modeling technology. The systematic and comprehensive understanding of large-scale biological systems such as the immune system requires developing multiscale models through integration of multiple modeling technologies as well as large and diverse data types. Immunological processes are studied today with advanced technologies at various spatial scales. For instance, imaging techniques and microscopy are used to identify tissue-level changes, flow and mass cytometry for extracting cellular-level differences, and RNA-seq, RT-PCR or microarray for gene-level variation. Utilization of such high-dimensional big and diverse data types calls for more comprehensive modeling approaches. Furthermore, studying biological phenomena at different scales often requires different modeling technologies. ENISI is a multiscale modeling platform that efficiently integrates multiple modeling technologies to investigate immunological mechanisms across spatiotemporal scales.

### Modeling technologies

Types of modeling technologies are diverse; however, in this study the focus is on equation-based and agent-based models. Equation based models are captured using mathematical equations, such as ordinary differential equations (ODE) and partial differential equations (PDE). ODEs can easily capture entity changes in time but not in space. PDEs can capture changes in both time and space but are more complex to solve. In general, the complexity of equation-based models is determined by the number of equations describing the model. Small numbers of equations can be analytically solved; however, large numbers of equations can only be solved numerically. Even though mathematical equations are often elegant and efficient representations, many biological phenomena can not be easily captured using this mathematical formalism.

An agent-based model, ABM, is comprised of agents and their interactions. Like objects in objected-oriented design, agents in ABMs can capture arbitrary complex knowledge. For example, agents can; i) have properties to represent different entity states, such as sex, genotypes, size and color, ii) be assigned to specific locations and move spatially, iii) interact with the environment and other agents, iv) be represented in a hierarchical structure. ABM is capable of modeling multiscale and highly complex biological phenomena; furthermore, ABM can also integrate multiple modeling technologies.

### Modeling tools

Computational modeling technologies cannot be separated from the modeling tools. Without user-friendly tools, modeling is a daunting task for scientists without extensive computational skills. A key feature of a practical and valuable multiscale modeling tool rests in its ability to assist biologically skilled scientists build useful multiscaled models to generate novel hypotheses.

Engineers can use Matlab to develop ODE-based models; however, computational biologists rely on tools such as COPASI [1] and Virtual Cell [2] due to their customized user-friendliness and usability features. COPASI provide user interfaces for defining equations, entities, and rate laws. Biologically skilled scientists with limited knowledge of mathematical equations can utilize COPASI to model complex networks. COPASI currently supports only ODE-based models. For agent-based modeling, there are several existing tools such as SIMMUNE [3] and Basic Immune Simulator, BIS [4]; however, these are not designed to be easily extended to developing multiscale models of enteric immune systems. For generic modeling framework, computational biologists uses NetLogo [5,6] or Repast [7]. In comparison, NetLogo has better development efficiency but Repast provides better flexibility and performance. Moreover, the high-performance computing (HPC) capability of Repast provides greater scalability.

### ENISI Visual and ENISI MSM

**Figure 1:**
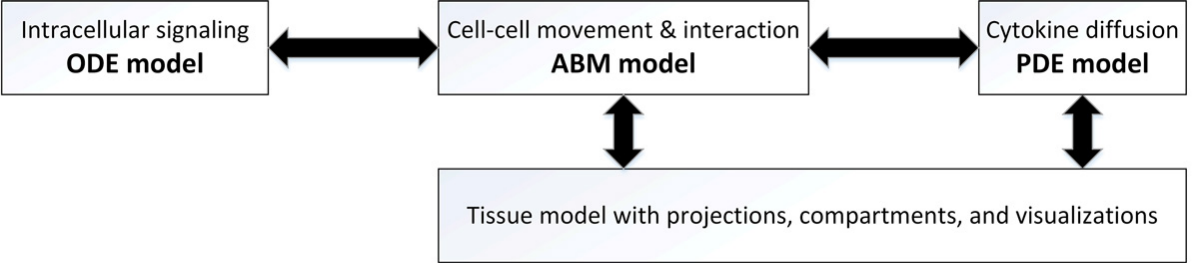
**ENISI MSM: A multiscale modeling platform integrating intracellular, cellular, and tissue scales and multiple modeling technologies**.

Development of ENISI, Enteric Immune Simulator, led to the development of a comprehensive model for enteric immune systems. ENISI can be used to develop multiscale models using ODE, PDE, and agent-based modeling frameworks. The resulting multiscale models include intracellular as well as intercellular scales and are able to represent signaling pathways, transcriptional regulation, metabolic networks, gene-regulatory networks, cytokine and chemokine diffusions, cell movement, tissue compartments simultaneously (Figure 1). In ENISI, intracellular signaling networks are modeled by ODEs; extracellular chemicals and proteins diffusions are modeled using PDEs; and the cell movements are modeled by agent-based models.

ENISI adopts object-oriented principal, i.e., entities on different scales are objects and these objects are hierarchically organized. During the development of ENISI, three versions were released; ENISE HPC [8] ENISI Visual [9] and ENISI MSM [10]. ENISI HPC focuses on scalability by implementing a parallel simulation framework, ENISI Visual focuses on visualizations, and ENISI MSM on the integration and performance matching among heterogeneous modeling technologies.

In the rest of this paper, related works are introduced before describing the scales, technologies and tools implemented in ENISI. Subsequently, the paper will focus on the technical challenges encountered during the system development, including the adoption of object-oriented design principals, the visualizations, and the performance matching techniques used for the integration of heterogeneous modeling technologies. The paper will present an empirical proof-of-concept study before discussing scope and limitations of the system as well as potential future directions

## Related work

Modeling in biology has long history tracing back to 1970s [11]. Early modeling techniques utilized a reductionist approach and models were largely based upon mathematical equations. With the introduction of computational systems biology [12,13] and the emergence of computation technologies, computational biology [14] and modeling techniques [15,16] have seen significant progress, including techniques such as equation-free modeling for describing dynamic systems [17] as a result of rapid increase in the computational power. In computational immunology, artificial immune systems (AIS) [18,19] have emerged as an independent research area across multiple disciplines, including mathematics, engineering, computer science, and immunology.

Computational modeling techniques can capture existing knowledge into models and discover new knowledge through model analyses and simulations. In this study, we focus on three popular modeling techniques in computational immunology, i.e., ODE, PDE, and agent-based [10,19-22]. Perelson *et al*. [23] presented ODE models for the dynamics among HIV virus and immune cells. Agent-based models can be powerful tools [24] for computational biology. Parunak *et al*. [25] compared ABM with equation-based models. Materi *et al*. [26] discussed computational modeling techniques, including ODE, PDE, and ABM, and tools used in drug discovery and development. The virtual cell [2] is a software environment for modeling a single cell using ODEs and PDEs.

In regard of multiscale models, identifying the appropriate linkages that facilitate integration of different models across scales is critical. Krinner *et al*. [27] coupled an agent-based model of hematopoietic stem cells with an ODE model of granulopoiesis and implemented this multiscale model in Matlab. Dwivedi *et al*. [28] presented a multiscale model of Interleukin-6-mediated immune regulation in Crohn disease and its application in drug discovery and development; the multiscale model was based on ODE. Hayenga *et al*. [29] argued that i) vascular systems are complex and require faithful multiscale models composing of sub models at all scales (macro, micro, and nano), and ii) efficiently coupling between sub models is critical for the performance of such models.

In a recent work, a multiscale modeling approach was utilized to identify the chemical, biological and mechanical mechanisms of scar formation and wound healing where cross-talk between the different fields could have a significant impact on would management and individualized care [30]. Furthermore, novel software workflow (EPISIM) is being developed for semantic integration of SBML-based quantitative models in multiscaled tissue models and simulation [31]. In essence, using EPISIM or similar tools it is possible to link cellular states such as differentiation to biochemical reaction networks such as lipid metabolism pathways. In addition, calibrated models can be integrated with a larger pool of reusable models available in the Biomodels database, which has over 163 metabolism themed models [32].

However, the challenges of modeling and specifically multiscale modeling are manifold, such as model complexity, large parameter space for model calibration, differences in time scales, cellular states as well as differences in technology used for the development of each individual scale. Hence, even though novel software workflow (such as EPISIM) are being developed for semantic integration of SBML-based quantitative models, the multiscale integration is still in its infancy. In ENISI MSM, deterministic and rule-based models are integrated in a unified fashion. Nonetheless, one of the key strengths of a multiscale modeling technology rests in its usability which would allow researchers with limited technical expertise build multiscale models.

Developing multiscale modeling tools could be achieved by programming the software in Java, C++ or any other programming language. In addition, there are also several existing agent-based immune simulators, including SIMMUNE [3], ParIMM [33], ImmSim [34], SIS [35], and NFSim [36], that have been developed over the past decade. For instance, SIMMUNE is a modeling environment where cell-cell and cell-molecule interactions could lead to an adaptive behavior that is context specific. SIMMUNE takes a generic approach and can be used to simulate a wide area of signaling cascades that may not directly relate to immunology. ImmSim is a very simple rule-based cellular automaton that was able to reproduce several phenomena in immunology. However, due to the lack of modularity and scalability, the needed effort to refine and expand a generic simulator to a specific field is considerable and requires extensive technical knowledge. Similarly, Basic Immune Simulator, BIS [4], and lymph node B cell simulator [37] are two additional examples of immune simulators that are developed using open source platforms. BIS was developed using Repast NetLogo [5,6], a popular ABM platform, and lymph node B cell simulator was developed using Rhapsody [38]. They both provide suitable animations. Additionally, Railsback *et al*. [39] surveyed several common platforms that could be used for the development of multiscale platforms, including Repast [7], Netlogo [5,6], and Swarm [40]. Macal *et al*. [41] presented comparisons of the development approaches and concluded that in general Netlogo and mathematic packages are easier to develop but provide less capabilities; Repast, on the other hand, is more involved and complex but it provides added benefits and can be more powerful. Furthermore, Matlab, an engineering programming language, is also widely used in computational modeling [27,42,43]. The latter has its limitations, including not being open source. COPASI [1] on the other hand is an open source software tool that is based on C++ but provides language bindings to python and Java; it is SBML-compliant and provide practical user interface (UI) for ODE-based models. COPASI can be used efficiently in the development of multiscale models that are modular and scalable.

The gastrointestinal tract has evolved to allow absorption of food and nutritional components required to sustain the organism and facilitate colonization of the mucosa by commensal bacteria while eliciting immune responses against pathogens. Gastroenteric bacteria including *Helicobacter pylori, Escherichia coli*, and *Clostridium difficile *can cause acute and chronic inflammations impacting worldwide populations. To better understand the impact of these pathogens on the immune system, and characterize the immune response, a systematic multiscale model of the gastrointestinal immune system that spans across tissue, cell, proteins and genes was developed [44,45]. Chakraborty *et al*. [46] reviewed several successful computational models in immunology and suggested that hierarchically accurate multiscale comprehensive models can be of great value for understanding the effect of i) drugs designed to correct pathologies, and ii) cellular and molecular level processes that could lead to effective self-tolerance to address the fight against tumors as well as chronic infections. Finally, more recently Sloot *et al*. [47] reviewed multiscale modeling in biomedicine and discussed some challenges.

Our previous work [8,48] has shown that implementation of ENISI using MPI achieves great scalability for up to 576 processing elements when simulating a population of 10 million cells. Also, we have demonstrated experience with ODE, SDE and ABM [21,49,50]. We have constructed a system of 29 ODEs representing dysregulated immune responses in IBD [51]. Building on our previous work, ENISI MSM [10] integrates COPASI, the ODE solver, ENISI, the agent based simulator and ValueLayer library from Repast, and the PDE solver to model cytokine and chemokine diffusion. COPASI [1], an ODE-based modeling tool, is widely used for computational biology for modeling "inside the cell" signaling/transcriptional networks inside the cell and performing steady-state and time course analyses in the ENISI MSM platform. ENISI Visual [9] is an ABM tool for simulating tissue-level immune responses and cell populations in the gut. ENISI allows design of multiple synthetic compartments, such as the lumen, epithelial barrier, or lamina propria; it can also simulate multiple types of immune and epithelial cells. The ENISI MSM prototype allowed a real time visualization of the simulation. ValueLayer, the PDE solver of our MSM platform, uses REPAST [52] a family of advanced, free, and open source Java-based ABM platform [7] as a reusable software infrastructure [53].

ENISI MSM [10] extended ENISI Visual and integrate COPASI based ODE [20] and SDE [54] models into ABM. ENISI MSM was able to address the limitations of previous ENISI tools by providing the capabilities to model at four orders of spatiotemporal scales in an integrated and seamless fashion.

ENISI is the first multiscale modeling platform that can couple ODE, PDE, SDE, and ABM models concurrently engineered to investigate mucosal immune responses. Table 1 shows the scales, their properties, modeling technologies and the tools used for each scale. This paper focuses on the design architecture and implementation challenges of ENISI, including object-oriented design principal, visualizations, and performance matching. Three performance coupling and matching techniques between different sub-models are also presented.

**Table 1:** The four scales of ENISI models, their spatial and temporal properties, as well as modeling technologies and tools for each scale.

Scale	Example scenarios	Spatial (m)	Time (s)	Technology	Tool
Intra-cellular	Signaling pathways	Nano	Nano	ODE	COPASI
Cellular	Cell movement and subtypes	Milli	Tens	ABM	ENISI
Inter-cellular	Cytokine diffusion	Milli	Tens	PDE	ValueLayer
Tissue	Inflammation and lesions	Centi	Thousands	Projections	ENISI

### ENISI: modeling scales, technologies, and tools

ENISI simulates gut mucosal immune responses. The gut immune system accounts for 70% of the human immune system. ENISI models four different scales: tissue, cellular, intercellular, and intracellular. ENISI architecture and its scales have been summarized in Figure 1 and Table 1. In the following sections, the implementation, modeling technologies and tools utilized for each scale are presented.

### Tissue scale

In the tissue scale, ENISI currently support modeling five different tissue types: the lumen, epithelium, lamina propria, draining lymph nodes, and blood (Table 2). ENISI tissues are implemented as compartments in two-dimensional spaces. Repast provides both grid and continuous spaces classes for further implementation. Grid spaces are useful to define neighbors while continuous spaces can be used to implement motion plans. ENISI models can implement multiple compartments in the grid; it can also provide boundaries between these compartments using the continuous spaces through the use of vertical lines, horizontal lines, or irregular shapes. The cells inside each tissue can move based on Brownian and chemokine-driven motions; these cells can also cross boundaries and move across different tissues.

**Table 2:** Compartments of the immune system modeled by ENISI.

Tissue type	Description
Lumen	The inner open space of a tubular organ such as the stomach or intestine.
Epithelium (Ep)	The thin monolayer of epithelial cells separating the lumen and LP. The epithelium is composed of several subsets of epithelial cells, but intraepithelial lymphocytes can also be present.
Lamina propria (LP)	The connective tissue underlying the Ep where most of the immune cells associated with the stomach mucosa reside. LP is an effector site.
Draining lymph nodes (LNs)	The secondary lymphoid organs draining the gastrointestinal tract. The LNs are inductive sites of the mucosal immune system; where immune responses are induced.
Blood	The source for the monocytes such as Macrophages, dendritic cells, and neutrophils.

### Cellular scale

ENISI simulates the following immune cell types: epithelial cells, macrophages, dendritic cells, neutrophils, B cells, T cells, and bacteria. The cells are modeled as agents in agent-based models. Each cell is an instance of an agent that has its own states and moves inside its designated compartments. Each cell type or agent is implemented as a class in Java. The Java cell objects can be placed inside a space in Repast and Repast simulation engine will execute the defined motion plan during each simulation cycle. The motion plan will determine the next location of the cell based upon the current location and the cell's speed.

The different immune cell types can have subtypes depending on the immune responses and their micro-environments as described below.

• Epithelial cells form the organism's first line of defense by preventing the entry of potentially dangerous microorganisms. Intestinal epithelial cells are continuously exposed to large numbers of commensal bacteria but are relatively insensitive to them. Following contact with pathogens they produce inflammatory mediators and anti-microbial peptides.

• Macrophages initiate the innate immune response against microbes following recognition of pathogen-associate molecular patterns through pattern recognition receptors. Following the phagocytosis of pathogens, macrophages present the antigens to T cells and produce different molecules, thus leading to the expansion and differentiation of lymphocytes. Depending on the environmental signals macrophages can differentiate into at least two different subsets, M1 ("classic" activation or pro-inflammatory) and M2 ("alternative" activation or anti-inflammatory). M1 macrophages are potent effector cells that produce pro-inflammatory cytokines while M2 macrophages counteract inflammatory responses and create an environment that promotes angiogenesis and tissue remodeling.

• Dendritic cells (DCs) are located at sites of pathogen entry in the gastrointestinal mucosa and are involved in the induction of effector and regulatory responses. Immature DC are professional antigen-presenting cells with the capacity to internalize and process pathogens, and present antigens via the MHC-class II pathway. Effector dendritic cells are professional antigen presenting cells with a role in inducing T cell-dependent effector responses such as T helper 1 (Th1) and Th17 responses. Tolerogenic DCs are a subset of DCs that mediate mechanisms of antigen specific tolerance induction in the periphery through induction of regulatory T cells (Treg).

• Neutrophils are part of the innate immune system and are highly motile. Neutrophils can be attracted by cytokines secreted by epithelial cells and macrophages and quickly move to the infected or inflamed areas. Neutrophils play a key role in defending against invading pathogens. They can recruit and activate other immune cells, phagocyte pathogens, and release soluble antimicrobials.

• CD4+ T cells are lymphocytes that mediate adaptive immune response. T cells usually are recruited by DCs and activate other immune cells such as B Cells and macrophages. There are several phenotypes of CD4+ T cells, including T helper 1 (Th1), T helper 17 (Th17), and regulatory T cells (Treg). Th1 cells represent an effector subset of CD4+ T cells involved in the cellular immune response and host defense against intracellular pathogens. They are centrally involved in cell-mediated immunity and the production of complement fixing antibodies. Th17 cells are a subset of effector T helper cells that produce interleukin-17 (IL-17) and exhibit effector functions such as clearance of pathogens, as well as involvement in lesions during immune mediated diseases such as inflammatory bowel disease. Treg cells are CD4+ T cells, which are critical for the maintenance of immune cell homeostasis.

• B cells are lymphocytes that play a major role in the humoral immune response. They produce antibodies against antigens, function as professional antigen-presenting cells (APCs), and eventually develop into memory B cells following activation by antigen interaction.

• Bacteria are prokaryotic microorganisms. There are approximately ten times as many bacterial cells in the human flora as there are human cells in the body, with large numbers of bacteria on the skin and as gut flora. The vast majority of the bacteria in the body are harmless, and some are even beneficial, for the immune and provide signals that facilitate tolerance and nutrition. However, a few species of bacteria are pathogenic and cause infectious diseases, including cholera, tuberculosis, dysentery, syphilis, anthrax, leprosy, and bubonic plague.

### Intercellular scale

Intercellular scale refers to cytokines and chemokines that are secreted by cells and diffuse in the gut tissue microenvironment and useful for engaging receptors on the cell surface and triggering signaling inside the cells. The cytokines, chemokines, and their change in concentration over time are modeled by PDE models. The PDE solver of ENISI MSM uses ValueLayer library of Repast Symphony [7]. The two main classes of the ValueLayer library are GridValueLayer and ValueLayerDiffuser. GridVaueLayer stores the values for a grid space and provide methods to manipulate the values for individual grid cells. ValueLayerDiffuser diffuses the values of the GridValueLayer according to the two constants: evaporation constant and diffusion constant. The evaporation constant determines the degradation value and the diffusion constant determines the migrations of values of a grid cell to its neighboring grid cells. The grid space could be modeled using two- or three-dimensional space.

Implemented in the ValueLayer library, the diffusion of cytokines and chemokines follows equation (1), where *vn *is the value of the grid cell itself at step *n*. The values of *ce *and *cd *are evaporation constant and diffusion constant respectively. The last part of the equation is the summation of the differences between all the neighboring cells and the cell itself.

(1) $$v_{n}=c_{e}*\left[v_{n-1}+c_{d}*\sum \left(v_{n-1}^{neighbor}-v_{n-1}\right)\right],$$

### Intracellular scale

Intracellular scale models the signaling reactions at the protein level inside each individual cell during the immune response. ODE-based models are used to represent the intracellular pathways. The model development and simulations are performed using COPASI [1]. COPASI, a widely used ODE-based modeling tool in computational biology, was originally designed for biochemical reactions. In addition, all the ODE reactions are first order and users can specify the rate of such reactions and change the parameters in the rate functions. COPASI was further expanded to model stochastic differential equations [54]. The three main steps for developing a COPASI model are summarized for the sake of clarity:

1. *Developing the network model*. Development of the first iteration of the model topology, which does not include dynamical properties, can be achieved using CellDesigner [55]. The initial model can then be imported into COPASI where additions can be made to the model. For instance, dynamical specifications can be added to all the ODEs for all the reactions.

2. *Calibrating the model*. Model calibration focuses on parameter estimation by fitting the simulations generated by the model with experimental data, extracted from literature or directly from wet-lab. COPASI provides a simple user-interface for the model calibration process.

3. *Performing analyses*. The types of analyses that can be performed in COPASI include metabolic, steady state, time course, and sensitivity etc. analyses.

### ENISI simulations

The multiscale model simulated in ENISI is executed in the following steps:

1. Initialization of simulated entities, including: i) the grid and continuous space; ii) cells, their subtypes, and their locations; and iii) value layers for inter-cellular cytokines.

2. During each simulation cycle the following actions are executed:

a. *Movement: *Cells move according to their movement plan and cytokines diffuse according to the diffusion constant.

b. *Computing and updating: *Each cell inspects its location from the projections, obtains cytokine concentrations from the ValueLayers, sends information to the ODE solver, and calculate its subtypes and cytokines that secret into the environment.

c. *Visualization: *The cell icon locations, the respective colors, and the grid cell background color will change during each simulation cycle.

### Development challenges

ENISI is implemented in Java and based upon the Repast Symphony. COPASI is written in C++; however, it provides a Java language binding which is instrumental in the development of this tool. The PDE solver library ValueLayer is part of Repast Symphony. Due to the hybrid modeling technologies we have encountered many challenges in developing ENISI, a multiscale modeling tool for computational immunology. In this section, the focus will be on three major challenges: 1) system design principle, 2) visualization, and 3) performance matching.

### Object-oriented design

Development of a multiscale modeling tool that incorporates multiple modeling technologies is challenging. It is therefore important to use a system design principle that will be able to integrate ODE, PDE, and ABM modeling technologies efficiently. Objected-oriented (OO) system design is widely used in software development for more than two decades. Objects in object-oriented design are similar to agents in agent-based modeling platforms. However, there are some fundamental differences, for instance, in agent-based modeling, an agent usually corresponds to a simulated entity, yet objects do not have such limitation in the OO systems. In the OO design, every entity is an objects. Each object can have data, set of properties, set of operations, and be associated with many other objects. For instance, a tissue is an object, it can have name, color, location, and concentration of chemicals etc. A cell can also be an object, it can have locations, mobility, phenotype, genotype, etc. A tissue can have many cells inside. A tissue can have methods to control the movement of cells. A cell can have methods to interact with the tissue object and neighboring cells. Furthermore, an ODE solver can be an object. If intercellular pathway networks are modeled using an ODE model, a cell can have an object of an ODE model and an object of an ODE solver. Hence, the ODE solver can take the ODE model as an input and compute the model simulation results. In fact, the object-oriented design principal is essential in the ENISI implementation. The OO design is the only principle that can consistently encapsulate heterogeneous concepts, entities, and relationship of multiscale models simultaneously in an efficient and modular manner.

ENISI MSM utilizes extensively object-oriented programming features such as encapsulations, inheritance, and polymorphism. Encapsulations: Objects can be used to encapsulate data and methods together. Many implementation details are encapsulated into objects and objects can represent a cell, a subcomponent such as an ODE sub model or a cytokine with its propagation and dispersion following PDEs. Inheritance and polymorphism: Common data and methods can be captured by a superclass that is inherited by multiple subclasses. For example, T cell can be represented by a super class and T cell subsets, such as Th1, Th2, Treg, Th9, or Th17, can be represented by sub classes. The same method can be implemented differently in the sub classes of epithelial and myeloid cells so that polymorphism can be achieved.

### Visualizations

ENISI simulates immune responses to enteric pathogens. It was developed based upon a popular ABM platform [56]. It simulates multiple compartments including lumen, epithelial, and lamina propia and multiple types of cells and microbiota components including epithelial cells, T cells, B cells, macrophages, neutrophils, dendritic cells, and bacteria. Each cell type can have several subtypes. For instance, T cells can be resting T cells, T Helper cells, or T regulatory cells.

To be able to control, observe, and adjust the simulations, ENISI provides interfaces for users to change simulating settings such as initial numbers of cells and simulation speed. ENISI provides real time simulation videos. Simulation snapshots and videos can also be saved as audio/video files. Cells are visualized as icons and they change colors when in different subtypes. The environment is represented as both 2-d grid spaces and also continuous space. Cells are moving and secret cytokines and chemokines into the tissue environment. The chemokines and cytokines diffuse in the environment and are visualized as background colors. In general, the inflammatory cell subtypes are represented in red colors and the regulatory cell subtypes in green colors. The same applies to the background colors. Red background indicates inflammatory cytokines while green indicates regulatory cytokines. The simulation results can be observed in real time. The simulation results can also be saved for further processing as tab-separated files.

### Tissues and compartments

**Figure 2:**
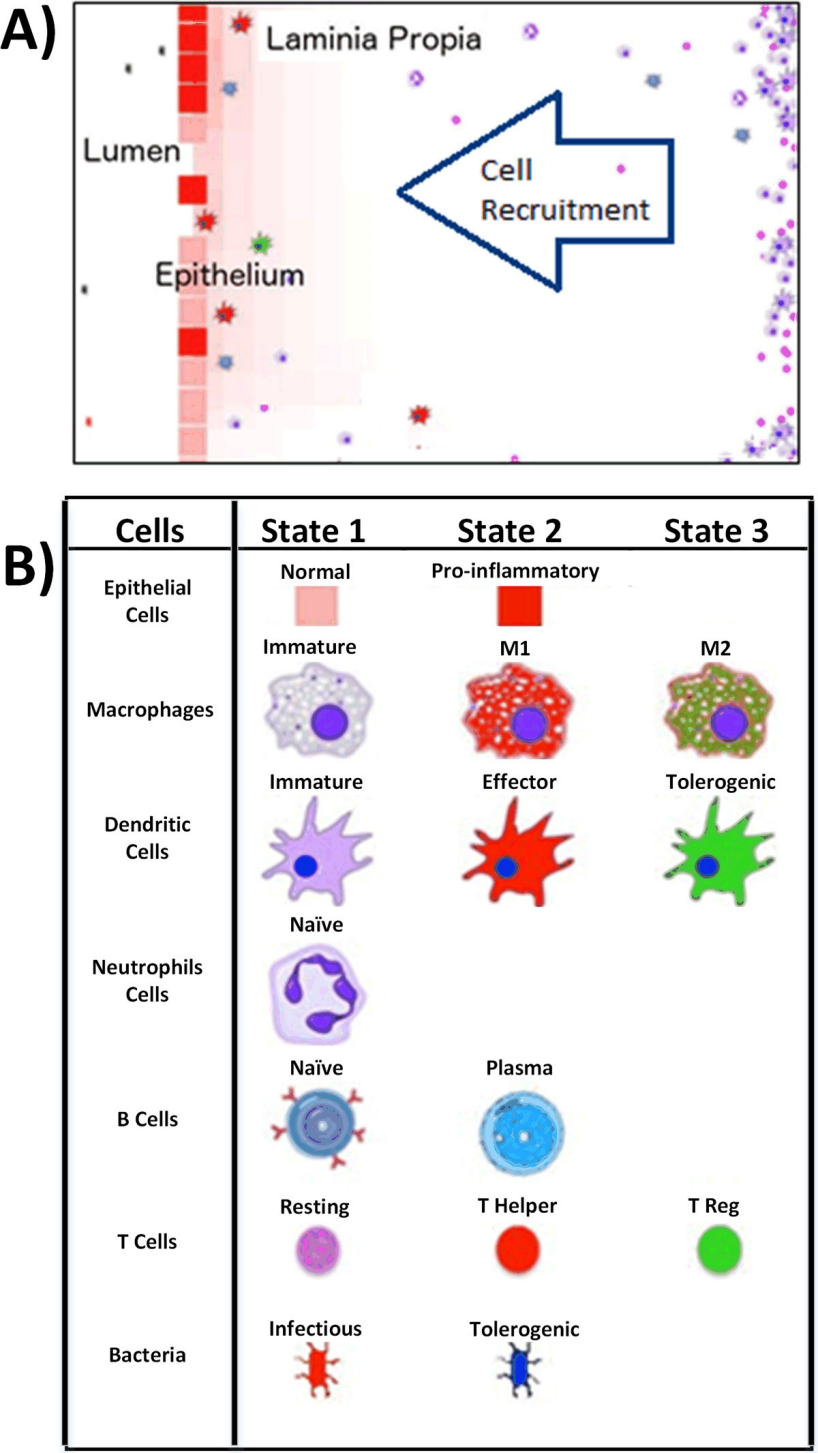
**ENISI compartments and cell types used in ENISI**. A) Different cells can move within or across compartment depending on their types and states. B) Agents, states, and symbols used in ENISI.

Figure 2A illustrates the three compartments of ENISI: lumen is on its left side, epithelium in the middle vertical layer, and lamina propria on the right side of the figure. The gastric lymph node and blood are not shown in this visualization. Both compartments can provide immune cells during the immune response. The recruitment of immune cells is represented by the influx of immune cells from the right side of lamina propia.

### Cell and cell state transition

Each cell has different states or phenotypes. For instance, an immature macrophage cell can become pro-inflammatory (i.e., M1) when in contact with pro-inflammatory T helper cells. In each simulation cycle, each cell inspects its neighbors and its environment and decides to either keep or change its state to an alternative state. Different cell types are represented by different symbols and the symbols change colors when the cells change functional types.

In general, with pro-inflammatory neighboring cells and pro-inflammatory cytokines, a cell has higher probability to change its state to pro-inflammatory. State transitions in this agent-based simulator are stochastic processes. The cell types, states, and symbols of ENISI Visual are presented in Figure 2B. In addition, all cells have dead states and their colors are black independent of their original state.

### Cytokine, chemokines, and microenvironment

ENISI users can add multiple cytokines and chemokines into a model by manipulating the concentrations of ValueLayers in the grid space. Some immune cells move depending on the concentrations of certain chemokines, while others change their internal states according to the cytokines presented in their microenvironment.

**Figure 3:**
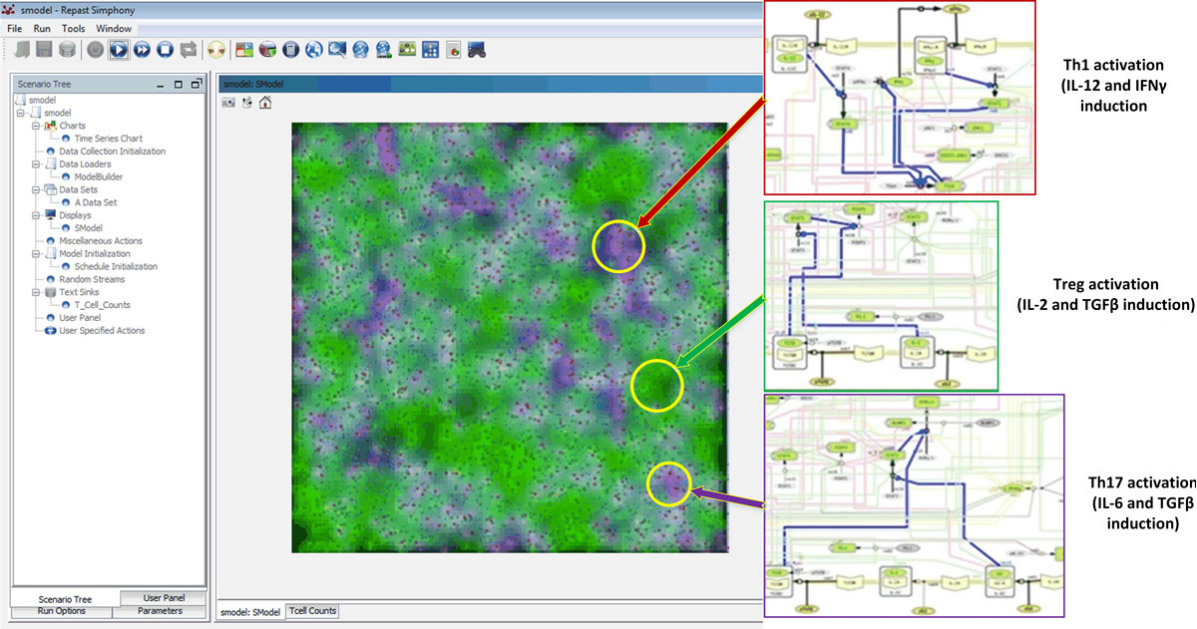
**ENISI user interface**. The left side is the control panels and users can set many simulation settings such as initial numbers of different cells, simulation speed, and ODE COPASI file path. The right side is the real-time simulation video with grids and icons of different colors. The regions highlighted correspond to Th1, Treg as well as Th17 activation.

The grid cell background color is visualized based upon the cytokine or chemokine concentrations. Currently, three cytokines can be visualized as three primary colors. Figure 3 shows the different patterns of background colors of grid cells. Color codes assigned to highlight the inflammatory response by shades of red and the regulatory response by shades of green.

### User interfaces, snapshots and animations

The interface allows users to control the initial cell concentrations, simulation outputs, and simulation speed etc. The users can also set batch simulation mode. Simulation outputs can be represented by animations, figures, and output data files. The data can be further processed through other data processing tools.

In addition to controlling the simulation speed, user can initiate, step, run, pause, or reset the simulation. Users can take snapshots and record videos. Figure 3 shows the ENISI interface, the right panel displays the simulation, and in the top panel are the control buttons while on the left side are the simulation settings. The windows can be dragged, repositioned, or relocated. Furthermore, additional windows can also be added to display complementary information from the simulation (such as diagrams of cell counts).

### Performance matching

Different scales have different spatiotemporal properties; therefore, performance tuning between layers is necessary. In this section, three techniques used for performing tuning are further discussed.

### Hybrid frequencies

The simple implementation of ENISI MSM calls all the sub-models in the different scales in each simulation cycle. If the cost of performing a sub-model simulation of scale s_i _is c_si_, then the cost for each simulation cycle will be approximately Σ_i _c_si _if the sub-model coupling cost is neglected as compared with the simulation costs of sub-models. However, different scales have different spatiotemporal properties and the frequencies of sub-model simulations can be different for different scales. Therefore, if the simulation frequency of scale s_i _is f_si _, i.e., the number of simulations performed in each simulation cycle, then the simulation cost of one cycle will be Σ_i _f_si _c_si _. For instance, if the simulation of one scale is performed once every 10 simulation cycles, then the frequency will be 0.1. In essence, the hybrid simulation frequencies across scales will significantly improve performance of a multiscale model.

### Optimal number of ODE solver objects

The number of projections and the number of cytokines tend to be small. For instance, in the empirical study section, the model has 2 projections and 6 cytokines. Each cytokine has one ValueLayer object and calls it to calculate the diffusions. On the contrary, one may have a large numbers of cells of different types, where each cell will call an ODE solver object to calculate the intracellular ODEs. If each cell is allocated with one dedicated ODE solver object, then the ODE solver needs to load the model file once and can remember all the status across the simulations. However, ODE solver COPASI object is a large object; therefore, loading millions of such objects in the memory will significantly slow the simulations.

Consequently, it will be more efficient if only one ODE solver COPASI object is implemented. The latter can be designed to serve all the cell objects, and implemented using the singleton design pattern. However, each time the COPASI object has to be reinitialized to the current settings of the cell, the latter has to be called. This process can significantly increase the computational load. Alternatively, one could keep a pool of ODE solver COPASI objects, which could have the added benefit of multi-thread environment. The optimized number of solver objects will depend on the hardware and software configurations such as memory size, CPU speed, number of cores etc. Furthermore, with the implementation of a parallelized HPC version of the ENISI MSM, the computational load will be distributed and considerably reduced, allowing the development and simulations of significantly large models, beyond unprecedented scales of 10^8 ^to 10^10 ^agents [8].

Therefore, one possibility is to have one ODE solver COPASI object that can serve all the cell objects; the latter can be implemented using the singleton design pattern. However, each time the COPASI object need to be re-initialize to the current settings of the cell that calls it; hence, increasing the computational load. Another option would be to keep a pool of ODE solver COPASI objects. This alternative will have benefits in multi-thread environment while balancing the memory and speed of the simulations. The optimized number of solver objects depends on for instance, the hardware and software configurations such as memory size, CPU speed and, number of threads.

### Model reduction

The CD4+ T cell differential model [20] is a comprehensive intracellular ODE-based model with 108 species, 46 reactions and 60 ODEs driving the activations and the inhibition pathways. If each naïve T cell calls this ODE model to calculate its subtype and determine the cytokines that it secretes, the computation cost will be astronomical. For developing a multiscale model, the comprehensive model was compressed into a reduced model with 9 species, 9 reactions, and 6 ODEs (see section on Empirical study). Alternatively, in cases where sufficient data are available, supervised machine learning techniques from artificial intelligence (AI) can be designed and optimized to replace the ODEs in the model. In fact, we have demonstrated [57-59] that Artificial Neural Networks (ANN) as well as Random Forest (RF) algorithms are efficient alternatives to ODEs and can reduce the complexity of intracellular network models by focusing on input and output cytokines. ANN and RF were optimized and evaluated using the CD4+ T cell differentiation model [20]; the models were also assessed by three published independent studies [60-62]. Because ODE-based modeling approaches require detailed knowledge about kinetic parameters, modeling using supervised learning methods can provide a realistic alternative when models are calibrated with experimental data. In the multiscale model development, it will often be necessary to modify the single layer sub-models before coupling them together into a multiscale model. The reduction in model complexity can be balanced with higher computational power for model simulations and more realistic number of agents for simulation studies.

## Empirical study

A proof-of-concept of multiscale model of gut inflammation was developed using the ENISI MSM system. This model can be used to run *in silico *simulations and computational hypothesis generation for further experimental validation; the model can also be used to test many hypothetical scenarios that are not possible to analyze with single scale models. Thus, the proposed approach facilitates connecting specific molecular events occurring inside the cell with major changes at the tissue level, such as changes of tissue architecture and immunopathologies occurring at the cellular and tissue levels. The multiscale model developed clearly demonstrates the capabilities of ENISI MSM as a multiscale modeling platform and the performance tuning benefits of the three proposed scale coupling techniques.

### ABM, ODE, and PDE sub-models

In the ABM model, we have bacteria, dendritic cells, and T cells that are implemented as three Java classes. The bacteria have three possible states/subtypes: dead, infectious, and tolerogenic. The dendritic cells have four possible states: dead, immature, effector, and tolerogenic. The T cells have five possible states: Th17, Th1, Treg, naïve and dead. Additional details regarding cell types and subtypes can be found in [9].

**Figure 4:**
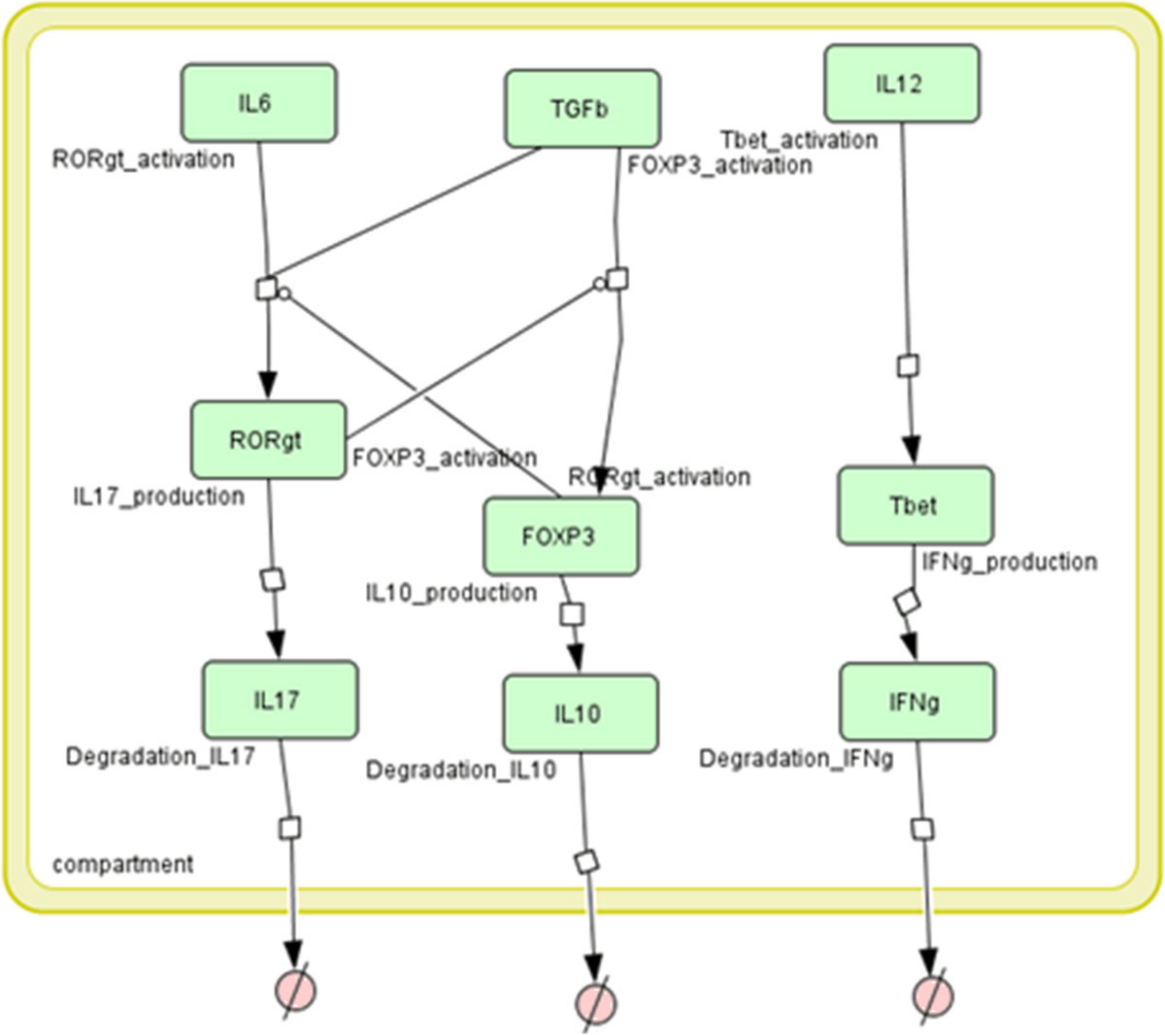
**The network of simplified CD4+ T cell differential model utilized for the development of the ODE model**.

The ODE model, implemented in COPASI, is a simplified version from the comprehensive CD4+ T cell differential model [20,49,63]. The network model, developed in CellDesigner, is shown in Figure 4. To summarize, naïve T cells will differentiate into Th1 cells and secrete INFγ into the micro-environment environment if cytokine IL-12 is positive. However, if TGF-β is positive and IL6 is negative, then the naïve T cells will differentiate into Treg cell and secretes IL-10 into the micro-environment. Furthermore, if both TGF-β and IL-6 are both positive, the naïve T cells will differentiate into Th17 cell and secret IL-17 into the environment. The COPASI ODE solver has a main object CCopasiDataModel that can load a COPASI model file for the initialization of the model. Following the initialization, the report and tasks are organized for further processing by the ODE solver. In addition, the ODE solver class also provide a hashMap for storing the concentrations of cytokines and chemokines. Those concentrations provide the initial values for entities in the model.

Six cytokines, three as inputs and three as outputs are modeled and implemented in this study. The three input cytokines are IL-12, TGFβ, and IL-6. The three output cytokines are INFγ, IL-17 and IL-10. The six ValueLayer objects are three evaporation constants, which are set to 0.98, and three diffusion constants that are set to 0.6.

### Model settings

The area in the model is defined as a square region with 100 ∗ 100 two-dimensional grid cells. At the start of the simulation, there are 1,000 bacteria, 50% infectious and 50% tolerogenic. Furthermore, there are 2,000 naïve T cells and 2,000 immature dendritic cells. The bacteria, T cells, and dendritic cells are evenly distributed in the square area at random. In one simulation cycle, these agents can move in a randomized fashion to any direction with an evenly distributed speed that can range between 0 and 1 grid cell side length.

When the immature dendritic cells (iDCs) meet with the infectious bacteria, (i.e. iDCs are in the same grid as infectious bacteria), the iDCs will differentiate into effector subtype. The effector dendritic cells (eDCs) will release IL-6 and IL-12 into the tissue micro-environment by setting the concentrations of the two cytokine value layer to 70, a relative value, at that grid cell. Alternatively, if the iDCs are co-located with tolegenic bacteria, they will differentiate into tolegenic dendritic cells (tDCs) and release TGF-β into the tissue micro-environment.

Since the 500 tolerogenic bacteria, 500 infectious bacteria, and 2,000 iDCs are randomly distributed in the area, some grid cells will have tDCs and some grid cells will have eDCs. In addition, as the cytokines are diffusing and evaporating, some grid cells will have only TGF-β positive, some will have both TGFβ and IL-6 positive. Therefore, naïve T cells will differentiate into Treg, Th17, or Th1 cells depending on their location and the cytokines present in those location.

Each naïve T cell will sense the concentrations of the cytokines in its grid cells and send those values to the ODE COPASI solver. The ODE solver will load the COPASI model file, set the input concentrations, and then perform the time course simulations. Ultimately the concentrations of the three output cytokines, i.e., IFNγ, IL-17 and IL-10, will be extracted and returned back to the T cell objects. Finally, the naïve T cell will differentiate into Th1, Th17, or Treg accordingly and the three cytokines will be released into the tissue micro-environment.

During each simulation cycle, the quantitative information will be visualized as described. The grid cell background color is visualized based upon the cytokine concentrations; the three cytokines are visualized as three primary colors. The color codes are designed to represent in red regions with higher Th1, in purple regions with higher Th17, and in blue regions with higher Treg cells. The intracellular ODE simulation results are displayed as texts in the terminal window for visual inspection during the simulation; the latter could be saved for further analysis.

### Simulation results

**Figure 5:**
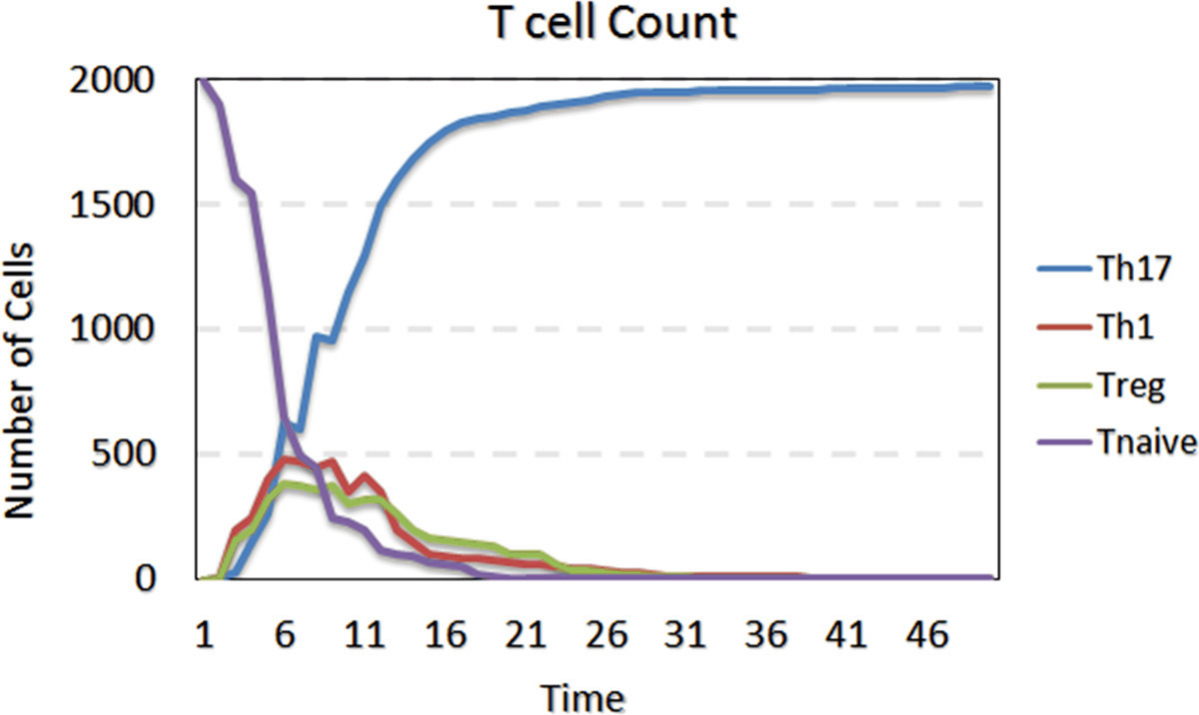
**T Cell counts in subtypes**. Simulation performed using the RM scenario. The X-axis is the simulation time in cycles and the Y-axis is the numbers of different T Cell subtypes. This figure shows the dynamics of T cell subtypes during the simulations.

The stochastic component of the model will lead to a situation where some areas in the grid will have higher concentrations of Th1, some Th17, and some will have a higher level of Treg cells (see Figure 3). Furthermore, Figure 5 represents a chart of T cell counts in subtypes; this representation highlights the temporal evolution of the immune response and the concentration of the different cell types over the course of the simulation. For instance, the concentration of Th1, Th17, and Treg cells are increasing in the initial stage of the simulation; however, after about 20 cycles, Th17 is the dominant cell type. This trend is due to the fact that the cytokines were isolated during the initial stage, and changes were made during the diffusion process and the inter-association and transformation of the agents in the later stages.

### Scale coupling and performance tuning

A single COPASI ODE solver object was used to generate the simulations with the CD4+ T Cell differentiation. During each cycle, simulations for all the three scales, cellular, intracellular, and intercellular, are performed. The performance, including initial CPU time for model initialization, CPU time for simulation, and memory footprint size, for this scenario (RM) is represented as the first row in in Table 3. The second scenario (BM) is implemented by replacing the reduced model with the comprehensive model in scenario one. The CPU time for 100 simulation cycles is significantly increased (> 75 times slower), showing that the comprehensive ODE model can be extremely inefficient even if it could provide more accurate results. Therefore, for running practical simulations it will be necessary to reduce the network before embedding it into multiscale models. In the third scenario (MS), each T cell object has its own ODE COPASI solver object. This scenario has longer initialization time; in fact initializing 2,000 ODE solver objects and loading 2,000 COPASI model files is extremely time consuming. In addition, the memory footprint is also significantly affected, an increase from about 400MB to 1.48GB. The MS scenario is based on the Reduced model (RM). The fourth scenario, dynamic frequency (DF), reduces the simulation frequency of the intracellular layer (ODE model) from 1 to 0.1. Hence, the ABM runs 10 simulation cycles and calls the ODE solver only once. The CPU time for simulation 100 cycles is therefore reduced significantly.

**Table 3:** The performance metrics of four simulation scenarios.

Scenario	Initial CPU Time (sec)	CPU Time for 100 simulation Cycles (sec)	Memory Footprint Size (MB)
Reduced model (RM)	14.35	296.81	399.1
Big model (BM)	14.17	23119.69	404.9
Multiple ODE solvers (MS)	5301.96	309.75	1480.0
Dynamic frequency (DF)	14.87	40.95	373.8

Initial CPU time is the CPU time used for the initialization of the simulation. In the RM, a reduced intracellular ODE model is utilized, where one single shared ODE solver object is used; the simulation frequencies are 1 for all scales. BM utilizes the comprehensive ODE model. MS is based on multiple ODE solver objects, one for each T cell object. DF uses reduced intracellular scale approach with simulation frequency from 1 to 0.1.

All the simulations are performed with a Mac Pro machine with Intel Core i7 2.7GHz 4-core CPU and 8GB memory. The performance metrics are measured using the activity monitor. The performance comparisons between the four scenarios are listed in Table 3. Further well-designed studies with better performance profiling are required to quantitatively investigate the scope and limitations of the three proposed scale coupling techniques. In this comparison, predictive power of the models should also be considered.

## Future work

ENISI is the first multiscale modeling platform for modeling mucosal immune responses. ENISI MSM has modular and coherent user interface and superior visualization. The system accelerates the development of comprehensive multiscale models by computational immunologists; in addition, ENISI accelerates the *in silico *experimentation process for hypothesis generation. It adopts an object-oriented design and can easily integrate entities at different scales. Furthermore, ENISI fuses heterogeneous modeling technologies that are suitable for different spatiotemporal scales. ENISI MSM fully integrates COPASI ODE models with agent-based models (ABM) to connect four levels of spatiotemporal scales. Three performance matching techniques were also analyzed.

An array of computational tools has been developed to address the urgent need of the scientific community [3,4,26,33,34,36,37]; however, the challenges of modeling and in particular multiscale modeling framework are manifold. ENISI addresses some of these challenges by adopting an integrated Object Oriented design principle. In addition, we are actively working towards further addressing these challenges by implementing the system using High Performance Computing technology. HPC-driven ENISI MSM will facilitate development of massively interacting models of the mucosal immune system and realistic high-resolution simulations with significantly larger number of agents (beyond 10^10^). The parallelized methods and higher computing power will be instrumental in the development of a scalable system. Additionally, as we have previously demonstrated [57-59] Artificial Neural Networks (ANN) as well as Random Forest (RF) algorithms are efficient alternatives to ODEs and can reduce the complexity of intracellular network models. we are working towards integrating machine-learning algorithms into ENISI platform for well-documented signaling pathways to increase scalability and performance. We are also improving the visualization component of the system by making the platform interoperable with Vislt [64]. In summary ENISI empowers experimentalists with a strong tool for computational modeling, thus facilitating fast and cost-effective knowledge discovery.

## Competing interests

The authors declare that they have no competing interests.

## Authors' contributions

YM, JBR, RH, SH and VA contributed in the original research ideas, developed ENISI MSM, and led the writing of this manuscript. VA, JBR, RH, and SH reviewed and edited the manuscript. AC helped develop the model used in the section of empirical study. XZ provided inputs and useful discussions for enteric immunity and immune responses. PL executed some of the simulations and provided some figures. CP provided useful input and discussions on the enteric immune responses. SH provided technical support for COPASI and reviewed the manuscript. JBR contributed in the original research ideas and reviewed the whole document.
